# Auricular Therapy to Control Pain in Women With Breast Cancer: Protocol for Systematic Review and Meta-Analysis

**DOI:** 10.2196/55792

**Published:** 2024-10-15

**Authors:** Ludmila Oliveira Ruela, Caroline de Castro Moura, Bianca Shieu, Yu-Min Cho, Chao Hsing Yeh, Franklin Fernandes Pimentel, Juliana Stefanello

**Affiliations:** 1 Ribeirão Preto College of Nursing University of São Paulo Ribeirão Preto Brazil; 2 Department of Medicine and Nursing Federal University of Viçosa Viçosa Brazil; 3 School of Nursing The University of Texas Health Science Center at San Antonio San Antonio, TX United States; 4 Cizik School of Nursing The University of Texas Health Science Center at Houston Houston, TX United States; 5 Department of Gynecology and Obstetrics Ribeirão Preto Medical School Breast Disease Division, University of São Paulo Ribeirão Preto Brazil

**Keywords:** breast neoplasms, cancer pain, pain, auricular acupuncture, auricular therapy, systematic review, meta-analysis

## Abstract

**Background:**

The increased incidence of breast cancer implies the appearance of frequent symptoms associated with disease and treatments, such as pain. For the management of this issue, auricular therapy has been used in a complementary manner, especially for its safety and analgesic action.

**Objective:**

This systematic review aims to summarize available evidence on the effects of auricular therapy on pain in women undergoing breast cancer treatment.

**Methods:**

This is a systematic review that includes randomized controlled trials that evaluated the effects of auricular therapy on pain in women with breast cancer, as compared with other interventions (sham or placebo auricular therapy, other nonpharmacological interventions, and routine pain treatments) during the treatment of the disease. Pain, whether induced or not by cancer treatments, is the main outcome to be evaluated. The search for the studies was performed in the following databases: MEDLINE through PubMed, CINAHL, CENTRAL, Embase, Web of Science, Scopus, VHL, TCIM Americas Network, CNKI, and Wanfang Data. The reviewers have independently evaluated the full texts, and in the near future, they will extract the data and assess the risk of bias in the included studies. The certainty of the evidence will be assessed using the Grading of Recommendations, Assessment, Development, and Evaluation (GRADE), and a meta-analysis will be carried out to evaluate the intervention, considering the homogeneity of the results, using the Cochran Q test and quantified by the Higgins inconsistency index. The guidelines of the PRISMA-P (Preferred Reporting Items for Systematic Review and Meta-Analysis Protocols) have been respected in the elaboration of this protocol.

**Results:**

The records screening stage has been completed, and the synthesis and meta-analysis were conducted in February 2024. We hope to have finished the preparation of the paper for publication by September 2024. Review reporting will follow standard guidelines for reporting systematic reviews. The results will be published in peer-reviewed scientific journals.

**Conclusions:**

This review will compile the strength of evidence for the use of auricular therapy in the management of pain in women with breast cancer during the treatment of the disease, identifying gaps in the available evidence as well as assisting health professionals in indicating the intervention for clinical practice.

**Trial Registration:**

PROSPERO CRD42022382433; https://www.crd.york.ac.uk/prospero/display_record.php?RecordID=382433

**International Registered Report Identifier (IRRID):**

DERR1-10.2196/55792

## Introduction

Pain is a symptom commonly associated with patients with cancer [1]. Considering breast cancer, the most common in the global female population [2], it is estimated that between 40% and 89% of this population will experience pain at some point during the disease [3,4], which is why it is important to consider effective therapeutic strategies for the management of this clinical condition.

Although there have been advances in the treatment of pain in patients with cancer [5], most feel pain at some moment of the disease [6]. This symptom may or may not be associated with antineoplastic treatments, such as chemotherapy, hormone therapy, and radiotherapy; have varied intensity; be acute or chronic; and generate physical and emotional distress that negatively impacts the patients’ quality of life [7-9]. Therefore, pain in patients with cancer demands special care from health care teams.

In that context, in recent years, complementary and alternative medicine (CAM) has gained ground in the area of oncology [10]. The desire to treat complications or cure cancer with holistic approaches, which influence health as a whole, enabling greater control over the treatment, is the main reason that leads these patients to seek these therapies [11]. On average, 51% of all patients with cancer use CAM, and this prevalence is even higher in women [11] with breast cancer [12,13].

There is a wide variety of therapies that encompass CAM, among which acupuncture is recognized as a complement to cancer treatment [14,15], especially breast cancer treatment [16]. Studies show that acupuncture is associated with the relief of disease-related adverse effects, such as pain [17-19].

Auricular therapy is a practice from traditional Chinese medicine, which includes, but is not limited to, acupuncture and acupressure [20,21]. According to the World Health Organization, auricular acupuncture is a microacupuncture capable of influencing the entire body [15]. This therapy was described in 500 BC in the classic text *Huang Di Nei Jing* and was later modified and updated by the French physician Paul Nogier in the 1950s [20,22].

Auricular therapy involves the relationships between the ear with the energy channels (the meridians) and the regions of the body through the theory of somatic reflexology, considering that diseases or symptoms of the body are projected onto areas of the ear [20,21]. Moreover, according to the perspective of traditional Chinese medicine, illnesses of the body are caused by an imbalance of energy (qi) in a person. Thus, auricular therapy treats health problems by stimulating points in the ear to equilibrate qi and activating the body’s meridians [21,23]. This stimulation can occur through different types of needles, seeds, spherical materials, electricity, lasers, and digital pressure [20,21,23].

The number of works involving auricular therapy has grown since the 1980s [23]. Studies show that this therapy is safe, effective, and cheap for the treatment of pain in people with cancer, revealing significant advantages in its application for the relief of this symptom [24-26]. In addition, auricular therapy can help to reduce analgesic consumption and treatment adherence [24]. When applied to women with breast cancer who experience pain, the therapy demonstrates benefits in reducing this pain, in addition to improving fatigue, sleep disorders [27], constipation [28], and quality of life [29].

Thus, considering that estimates point to a significant increase in the number of cases of breast cancer and a consequent increase in cases of pain in this population [30], auricular therapy can be a safe, inexpensive, and effective complementary treatment option for that complaint. However, the effects of this intervention on pain relief, specifically in women with breast cancer, are not fully elucidated in the literature. Therefore, this investigation is necessary.

Although previous reviews have evaluated the effects of auricular therapy in patients with cancer, particularly in relation to cancer pain [18,25], the effectiveness of this intervention, specifically in women with breast cancer, is still not completely clear, as studies have included populations with different types of malignant neoplasms.

To the best of our knowledge, no previous review has evaluated the effects of auricular therapy on pain management in women with breast cancer. Thus, our objective will be to identify available evidence on the effects of auricular therapy on pain in women undergoing breast cancer treatment. In addition, we intend to evaluate (1) the effects of this auricular therapy on pain intensity, (2) the risks and safety of auricular therapy in the treatment of pain in women with breast cancer, (3) whether there are auricular therapy protocols for this clinical condition, (4) the profile of professionals who apply the therapy, and (5) at which levels of care this treatment has been offered.

## Methods

### Protocol and Registration

The protocol of this review is registered in the PROSPERO (CRD42022382433) and has been developed according to the PRISMA-P (Preferred Reporting Items for Systematic Review and Meta-Analysis Protocols) guidelines [31-33]. The PRISMA (Preferred Reporting Items for Systematic Reviews and Meta-Analyses) guidelines [34] will be used to disclose future results.

### Development of the Research Question

The Population, Intervention, Comparator, and Outcomes strategy was used to develop the research question [35] (Textbox 1).

**Research questions were developed according to the Population, Intervention, Comparator, and Outcomes strategy.**

**Population, Intervention, Comparator, and Outcomes and their components**

- Research question
  - What is the efficacy of auricular therapy in controlling the pain induced or not by antineoplastic treatment in women with breast cancer, as compared with sham auricular therapy (a simulated intervention that may not be physiologically inert, as it stimulates tissue and the peripheral and central nervous system, even at minimal doses, such as nonspecific or superficial needling), or the treatment of routine pain or other nonpharmacological interventions?
- Population
  - Women with breast cancer undergoing cancer treatment
- Intervention
  - Auricular therapy with seeds, metallic points, magnetic points, plastic points (crystals), radionic crystals, needles (semipermanent and systemic), stiper, electrostimulation, laser therapy, and chromotherapy
- Comparator
  - Sham or placebo auricular therapy [36], other nonpharmacological interventions, and routine treatment
- Outcomes
  - Pain control induced or not by cancer treatment and adverse events originated from the uses of auricular therapy as a pain treatment for women with breast cancer

### Eligibility Criteria

#### Types of Studies

Studies that are a randomized controlled trial of auricular therapy to treat pain in women with breast cancer were included.

#### Types of Participants

The participants included women, 18 years of age and older, with breast cancer, regardless of the type of tumor and staging, including those in palliative care, and experiencing pain as adverse effects induced by cancer treatment or unrelated to the treatment.

#### Types of Interventions/Exposure and Comparison

Studies using auricular therapy as an intervention with any kind of device (mustard seeds or Vaccaria, metallic points, magnetic points, plastic points [crystals], radionic crystals, needles [semipermanent and systemic], stiper, electrostimulation [electroacupuncture, hai hua], laser therapy, and chromotherapy) were included. The intervention may be used by itself or in combination with other methods. The control included (1) sham or placebo auricular therapy or placebo, (2) conventional routine treatment with conventional drugs, and (3) other nonpharmacological interventions.

#### Types of Outcomes

The primary outcome was the intensity of pain presented during cancer treatment, evaluated through the Numerical Rating Scale, the Visual Analog Scale, and the McGill Pain Questionnaire, among other tools that may appear in the studies. The secondary outcomes, such as one’s quality of life, anxiety, and depression, among others, were also identified and evaluated.

Another variable investigated in the selected study outcomes was the possible adverse effects of using auricular therapy.

#### Setting

The study setting was public or private health care services.

#### Information Sources

The literature search was performed using MEDLINE through PubMed, CINAHL, CENTRAL, Embase, Web of Science, Scopus, VHL, TCIM Americas Network, CNKI, and Wanfang Data. No time and language restrictions were applied. Abstracts and full papers were obtained for each selected study based on the inclusion criteria. Additional studies were identified through the reference list of included papers and other systematic reviews that evaluated the effects of acupuncture on pain in people with cancer. The search for gray literature was performed on Google.

#### Search Strategy

To determine eligible studies for inclusion in the review, 2 reviewers independently assessed the English titles and abstracts of the papers; another 2 reviewers independently assessed the Chinese titles and abstracts of the papers. The search strategy was developed with the contribution of a librarian. We used Medical Subject Headings, keywords, and free text search terms. The key search terms were as follows: (“Acupuncture, ear” OR “Ear acupuncture” OR “Auriculotherapy” OR “Auricular therapy” OR “Ear therapy” OR “Ear therapies” OR “Auricular acupuncture” OR “Ear acupressure” OR “Auricular acupressure” OR “Auricular plaster” OR “Auricular electroacupuncture” OR “Ear electroacupuncture” OR “Laser auriculotherapy” OR “Auricular point*” OR “Ear point*” OR “Acupoint*”) AND (“Breast neoplasms” OR “Breast neoplasm” OR “Breast cancer” OR “Breast tumor*” OR “Breast tumors” OR “Breast carcinoma” OR “Breast diseases” OR “Mammary cancer” OR “Mammary tumor” OR “Mammary carcinoma”). The Chinese literature search terms were “疼痛,” “乳癌,” and “耳穴.”

### Study Records

#### Data Management

Searches in the databases were uploaded into the Covidence [37] to aid in the selection and management of papers. This software was used to manage the references obtained through database searches, the inclusion and exclusion of papers, and the removal of duplicates.

#### Selection Process

The selection of the papers was performed by 4 authors independently. Initially, the titles and abstracts of the papers were read considering the eligibility criteria. The preselected papers were then read in their entirety and evaluated for eligibility to confirm inclusion in the review. When there was disagreement between the authors regarding the eligibility of the paper, a third author was called, and the 3 authors came to a consensus regarding the inclusion or exclusion of the paper. The review selection process will be presented through the PRISMA flow diagram.

#### Data Collection Process

Data from the included studies will be extracted by 4 independent authors. To ensure consistency of the interpretation among these authors, they will undergo initial training. The differences found in the data extracted from studies will be resolved through discussion between these 2 authors and, in case of disagreement between them, a third author will aid in the decision. One of the authors will cross the data extraction forms in order to create a final single form, filled out completely with the data.

### Data Items

We prepared a form for data extraction, which will be finalized after the consensus of all authors. This form will extract data from the included studies (Table S1 in Multimedia Appendix 1). The intervention data will be extracted based on the methodological guideline for evaluating clinical trials with acupuncture STRICTA (Standards for Reporting Interventions in Clinical Trials of Acupuncture) [38].

### Risk of Bias in Individual Studies

The Cochrane Collaboration Risk of Bias Tool [39] will be used to evaluate the risk of bias in the included studies. Methodological quality will be assessed by 2 independent authors considering random sequence generation, allocation concealment, blinding of participants and personnel, blinding of outcome assessment, incomplete data results, selective reports, and other biases. Each of these domains will be rated based on the risk of bias as low risk, high risk, and unclear risk. Methodological biases identified by STRICTA will be related to the intervention bias. Disagreements regarding the risk of bias will be discussed and resolved by another author.

### Data Synthesis and Analysis

The meta-analysis will be carried out using the program R (version 4.2.2; R Foundation for Statistical Computing) with the package *metafor*, considering the evaluations carried out before and after the intervention. Dichotomous and continuous variables will be estimated, respectively, as relative risk and mean difference, with 95% CI. For missing data, an attempt will be made to contact the corresponding authors, twice at most, with an interval of 15 days between them in order to recover them. To describe the percentage of variation among studies due to heterogeneity, the Cochran Q method and the Higgins method (*I²* statistics) [40] will be used. *I²* with values of 25%, 50%, and 75% will be considered of low, average, and high heterogeneity, respectively [41]. If the heterogeneity presents high values (*I²*>50%), the authors will reach a consensus on the inclusion or exclusion of the meta-analysis in the study. The result will be presented through the forest plot [42]. If the meta-analysis is not included in the review, the selected studies will be presented through a systematic narrative synthesis.

### Certainty of Evidence

We will use the Grading of Recommendations, Assessment, Development, and Evaluation (GRADE) to evaluate the certainty of evidence [43]. Two authors will carry out this evaluation independently. When there is a disagreement between them, a third author will be consulted to resolve the disagreement.

## Results

The systematic review is ongoing. On June 11, 2023, we identified 1309 records, and after title and abstract screening, 47 reports were assessed for eligibility. The risk of bias analysis and meta-analysis were completed in February 2024, and we plan to release the results of the study by submitting the paper to a peer-reviewed journal in September 2024.

## Discussion

### Overview

An increase in pain complaints in women with breast cancer is expected in the upcoming years, given the growing incidence in the number of cases of this disease. As a result, more effective, cheaper, and safer treatments will be a priority in health services that involve this type of health care. In this context, auricular therapy can be a complementary option for pain management. However, the evidence for its use in controlling this symptom in women with breast cancer is not fully elucidated. In addition, the lack of protocols and knowledge about the profile of professionals who work with this practice, as well as the level of health care in which it is offered, can make it difficult for patients to adhere to and use the therapy in clinical practice. Therefore, we believe that this review is extremely important, as it will provide a comprehensive review of studies already published on the subject, identifying gaps in this knowledge and presenting the evidence available to date.

### Conclusions

This systematic review will provide evidence for the use of auricular therapy in the management of pain in women with breast cancer during the treatment of the disease. We believe this is an important question to assist health professionals in recommending and using the intervention in clinical practice, as well as benefiting the patients. Its results may provide direction for future studies.

## Appendix

Multimedia Appendix 1 Data extracted from the included studies.

## Abbreviations

CAM: complementary and alternative medicine
GRADE: Grading of Recommendations, Assessment, Development, and Evaluation
PRISMA: Preferred Reporting Items for Systematic Reviews and Meta-Analyses
PRISMA-P: Preferred Reporting Items for Systematic Review and Meta-Analysis Protocols
STRICTA: Standards for Reporting Interventions in Clinical Trials of Acupuncture

## Data Availability

Data sharing is not applicable to this paper as no data were generated or analyzed for this research protocol. Data extracted from the literature and generated during the systematic review will be included in the published paper or made available upon request.
