# Supplementary material for: Auricular Therapy to Control Pain in Women With Breast Cancer: Protocol for Systematic Review and Meta-Analysis
Source: JMIR Res Protoc. 2024 Oct 15;13:e55792. doi: 10.2196/55792 (PMC11522657; doi:10.2196/55792)
Supplement: Multimedia Appendix 1 [file resprot_v13i1e55792_app1.docx]

**Table S1.**

| Authors  Year of publication |  |
| --- | --- |
| Methods |  |
| Participants |  |
| Antineoplastic treatment |  |
| Intervention details |  |
| Control details |  |
| Outcome measures |  |
| Results |  |
